# Phenotypic and genotypic characterization of virulence markers and antifungal susceptibility of oral *Candida* species from diabetic and non-diabetic hemodialysis patients

**DOI:** 10.1186/s12903-023-02970-8

**Published:** 2023-05-04

**Authors:** Faezeh Mohammadi, Maliheh Charkhchian, Monirsadat mirzadeh

**Affiliations:** 1 Medical Microbiology Research Center, Qazvin University of Medical Sciences, Qazvin, Iran; 2 Department of Medical Parasitology and Mycology, School of Medicine, Qazvin University of Medical Sciences, Qazvin, Iran; 3 Metabolic Diseases Research Center, Research Institute for Prevention of Non-Communicable Diseases, Qazvin University of Medical Sciences, Qazvin, Iran

**Keywords:** Antifungal susceptibility, *Candida* species, Oral candidiasis, Biofilms, Virulence factors

## Abstract

**Background:**

Patients with chronic kidney disease undergoing hemodialysis are often colonized by *Candida* species with high possibility of fungal infections. The purposes of this study were to determine the prevalence of *Candida* species, evaluate antifungal susceptibility profile, biofilm formation, proteinase and phospholipase activities, and the frequency of virulence genes in the *Candida* species isolated from the oral mucosa of hemodialysis diabetic (DM) and non-diabetic (non-DM) patients.

**Methods:**

This study identified several species of *Candida* isolated from 69 DM and 58 non-DM patients on hemodialysis using phenotypic methods and PCR–RFLP technique. The identification of *C. albicans* and *C. glabrata* complex was performed by HWP1 gene and four oligonucleotides (UNI-5.8S, GLA-f, BRA-f, and NIV-f), respectively. Antifungal susceptibility to amphotericin B, fluconazole, itraconazole, voriconazole, and caspofungin was assessed according to CLSI M27-A3/S4. The biomass, metabolic activity of biofilm, proteinase (P_rz_), phospholipase (P_z_), and molecular study for virulence genes were assessed using crystal violet, XTT assay, agar-based hydrolytic enzyme, and PCR technique, respectively.

**Results:**

*Candida* prevalence was 44.9% with 47.8% and 41.4% among DM and non-DM patients, respectively (*P* = .045). The species identified were *C. albicans* (49.5%), *C. glabrata* (16.5%), *C. tropicalis* (12%), *C. kefyr* (8.8%), *C. parapsilosis* (6.6%), *C. dubliniensis* (3.3%), and *C. lusitaniae* (3.3%). The antifungal susceptibility profile showed that all *Candida* isolates were sensitive to amphotericin B, itraconazole, voriconazole, and caspofungin whereas fluconazole resistance was observed in 6.3% (MIC ≥ 64 μg/mL) of *C. albicans* and 6.6% of *C. glabrata* (MIC ≥ 64 μg/mL). The susceptible- dose-dependent rate was found in 10.5% of *C. albicans*. The P_rz_ values of *C. albicans* ranged from 0.37 to 0.66 for the DM and 0.44–0.73 for the non-DM group (*P* < 0.05). The non-*albicans Candida* (NAC) species produced higher degree of biomass and metabolic activity compared to *C. albicans* (*P* < 0.05). Furthermore, significant (*p* < 0.05) correlations were detected between the biofilm formation with P_rz_ values and fluconazole MICs. The most detected virulence factors were ALS3 and Sap5.

**Conclusions:**

These results showed the importance of prevalence of NAC species in hemodialysis patients. Investigating antifungal susceptibility profile made a better understanding of the role of virulence markers in the pathogenesis of *Candida* strains.

## Background

Chronic Kidney Disease (CKD) results from progressive and irreversible damage to kidney function that leads to uremia [1]. The leading causes of CKD in adults are diabetic nephropathy and high blood pressure [2]. Research shows that salivary glucose levels are significantly higher in patients with diabetes than in healthy non-diabetics, which is also involved in *Candida* colonization. Pseudomembranous candidiasis, prosthetic stomatitis, erythema, and hyperplastic candidiasis are the clinical symptoms of oral candidiasis (OC) [3]. The most common responsible pathogen is *Candida albicans*, followed by non-*albicans Candida* (NAC) species [4, 5]. The *Candida* species such as *C. albicans*, *C. parapsilosis* and *C. glabrata* are considered as “cryptic species complexes” [6]. The correct identification of these *Candida* species is important due to the difference in virulence and antifungal resistance. Therefore, distinguishing between closely related *Candida* species is not possible by phenotypic methods and requires developed molecular techniques [7]. Different levels of antifungal resistance may occur among *Candida* species. Recent studies have shown increasing level of fluconazole resistance. The high resistance of *Candida* species to antifungals can challenge the management of CKD patients [8]. Extracellular hydrolytic enzymes are associated with increased colonization and invasion of host tissue. Secreted aspartyl proteinases are encoded by a family of 10 Sap genes. These genes can digest host proteins as a nitrogen source [9]. Production of phospholipase causes cell membrane disruption and hydrolysis of phospholipids. Among the four different classes of phospholipases, only B1, and D1 play the most important role in fungal pathogenicity [10]. A biofilm is a complex of microorganisms surrounded by an extracellular matrix. *Candida* biofilm is composed of a thick network of yeast cells, pseudohyphae, and hypha [11]. Agglutinin-like sequence (ALS) gene family is mainly responsible for adhesion and biofilm formation in *Candida* species [12]. Additionally, the hyphae wall protein produced by the HWP1 gene, affects yeast cell adhesion and regulates *C. albicans* biofilm formation [13]. The purposes of this study were to determine the prevalence of different *Candida* species isolated from the oral mucosa of diabetic and non-diabetic patients with CKD undergoing hemodialysis in Qazvin, Iran, and also to investigate antifungal susceptibility, level of hydrolytic enzymes secretion, biofilm activity, and finally the pattern distribution of virulence genes of *Candida* species.

## Methods

### Study population

In a cross-sectional study, the oral mucosa samples of 127 patients (69 diabetic (DM) and 58 non-diabetic (non-DM) CKD patients, admitted to the Hemodialysis Unit at Qazvin BooaliSina Hospital, Iran, was examined during a period between 2019 and 2021,. Demographic data, kidney disease profile, history of diabetes mellitus (DM), and clinical laboratory reports were collected from the patients’ clinical records. Informed consent was delivered by all patients for sampling from oral cavity. The exclusion criteria were lack of patient contentment, unstable clinical conditions, and a hemodialysis history of less than three months. The diagnosis of OC was made according to the clinical criteria presented by Holmtup and Axel [14]. Exfoliative cytology and Periodic acid-Schiff (PAS) staining were performed to confirm OC infection.

### Collection of samples

The subjects were asked to refrain from eating, drinking, smoking, or chewing gum for at least 60 min. The samples were collected by washing the oral cavity with 10 ml of sterile distilled water for 30 s and transferred to the medical mycology laboratory of Qazvin University of Medical Sciences. The samples were centrifuged at 2000 rpm for 15 min and the supernatants discarded. The sediments were inoculated onto Sabouraud dextrose agar medium (SDA, Difco) plates containing chloramphenicol and incubated for 48 h at 37 °C. *Candida* species were identified using germ tube formation (2 h at 37 °C in serum), chlamydospore production on Corn Meal Agar (CMA), colony color on CHROMagar Candida medium a (CHROMagar, Paris, France), and molecular technique (RFLP-PCR).

### Molecular identification of *Candida* Species

#### PCR–RFLP profile

For molecular identification of *Candida* species, the PCR–RFLP profiles were used. Briefly, the contiguous ITS1-5.8SrDNA-ITS2 region was amplified using PCR mixture containing 12.5 µl of 2 × Red PCR master mix, 10 pmol of each ITS1 (5′-TCCGTA GGT GAA CCT GCG G-3′) and ITS4 (5′-TCC TCCGCT TAT TGA TAT GC-3′) primers, 2 µl of extracted DNA in a final volume of 25 µl. The amplification program was as follows: initial denaturation at 94 °C for 5 min, followed by 35 cycles of denaturation at 94 °C for 30 s, annealing at 56 °C for 45 s, and extension at 72 °C for 1 min, with a final extension step at 72 °C for 7 min. The restriction fragment was then obtained by digestion of 10 μl PCR products with 0.5 µl of restriction enzyme *Msp I* (Fermentas, Vilnius, Lithuania). The PCR products and digested PCR products were visualized by ethidium bromide in 1.5 and 2% agarose gel electrophoresis, respectively.

#### Amplification of the HWPI gene and the 5.8-S ribosomal RNA gene

Identification and differentiation of cryptic species within the *C. albicans* complex were performed by PCR amplification of the hyphal wall protein 1 (hwp1) gene by using the forward 5′GCTACCACTTCAGAATCATCATC-3′ and reverse 5′ GCACCTTCAGTCGTAGAGACG-3′ primer pairs. The amplification program was as follows: initial denaturation at 95 °C for 5 min, 30 cycles of denaturation at 94 °C for 45 s, annealing at 58 °C for 40 s, extension at 72 °C for 10 min, and final extension at 72 °C for 10 min. The PCR products were separated and visualized on a 1% agarose gel. The amplified products were separated by electrophoresis on agarose gel. The different sizes generate by three species in the *C. albicans* complex: *C. albicans* (~ 1000 bp), *C. dubliniensis* (569 bp), and *C. africana* (~ 700 bp). The definite identification of *C.glabrata* complex (*C.glabrata*, *C. nivariensis* and *C. bracarensis*) were performed by PCR amplification of the 5.8-S ribosomal RNA gene. The reaction mixture in a 25 µL volume contained: 2 × Red PCR master mix, 10 pmol of each oligonucleotide (UNI-5.8S 5’-ACCAGAGGGCGCAATGTG-3’, GLA-f 5’-CGGTTG GTGGGTGTTCTGC-3’, BRA-f 5’-GGGACGGTAAGTCTCCCG-3’, NIV-f 5’-AGGGAGGAGTTTGTATCTTTCAAC-3’), and genomic. DNA. Conditions for multiplex-PCR were as follows: initial denaturation step at 95 °C for 5 min, followed by 34 cycles of 30 s at 94 °C, 40 s at 60 °C, and 50 s at 72 °C, and a final extension step of 10 min at 72 °C. The expected DNA fragment were 397, 293 and 223-bp for *C. glabrata *sensu stricto, *C. nivariensis*, and *C. bracarensis*, respectively.

#### Antifungal susceptibility testing

The minimum inhibitory concentrations (MICs) were determined through broth microdilution, according to the guidelines M27-A3/S4 of the Clinical and Laboratory Standards Institute performed in 96-well plates for amphotericin B (AMB), fluconazole (FLC), itraconazole (ITC), voriconazole (VRC), and caspofungin (CAS). The final concentrations of the tested antifungal agents ranged from 0.032 to 16 µg/mL for AMB, ITC, and VRC, 0.032 to 64 µg/mL for FLC, and 0.008 to 8 µg/mL for CAS [15, 16]. The final yeast suspensions of fresh colonies were added to each well, except for the negative control well. The plates were incubated at 35 °C for 24 h and MICs were determined visually. Minimum inhibitory concentration (MIC) was determined according to the guidelines. Minimum Inhibitory Concentrations for FLC, ITC, VRC, and CAS were described as the lowest concentration of the drug that could reduce fungal growth by 50% to 90%, compared to the growth in the control well. The MIC for AMB was described as the lowest concentration of the drug that could inhibits visible yeast growth completely.

#### Proteinase activity

Proteinase activity (P_rz_) was measured using bovine serum albumin (BSA) method [17]. A yeast suspension was prepared from each strain using the Yeast extract peptone dextrose (YEPD) medium. Then, 10 μl of this suspension (0.5 McFarland turbidity) was plated on medium containing 0.04 g MgSO4.H2O, 0.5 g K2HPO4, 1 g NaCl, 0.2 g yeast extract, 4 g glucose, and 0.5 g BSA (pH = 5.0). All plates were incubated at 37 °C for 6 days. The zone of proteolysis around the colonies of each isolate was calculated through the ratio of colony diameter to colony diameter plus the diameter of the clear zone (mm) around the colony. The P_rz_ values were considered as follows: P_rz_ = 1 as negative production; P_rz_ between 0.9 and 1, ( +); P_rz_ between 0.89 and 0.80, (2 +); P_rz_ between 0.79 and 0.70, (3 +); and P_rz_ less than 0.69, (4 +). Examination of the activity of both extracellular enzymes was repeated twice. *C. albicans* ATCC 10231 was used as a control strain.

#### Phospholipase activity

The phospholipase activity (P_z_) of the *Candida* isolates was evaluated using the egg yolk agar [18]. The suspension (0.5 McFarland turbidity) was placed on the surface of SDA medium (pH = 4.3) containing 1 M NaCl, 5 mM CaCl2, and 8% sterile egg yolk emulsion and plates incubated at 37 °C for 4 days. Phospholipase activity was calculated similarly to what was mentioned above for proteinase activity. Examination of the activity of both extracellular enzymes was repeated twice. *C. albicans* ATCC 10,231 was used as a control strain.

#### Biofilm biomass quantification

The crystal violet assay (CV) was used to quantify the total biofilm biomass [19]. In brief, the fresh colonies of *Candida* isolates were transferred to Sabouraud dextrose broth (SDB) medium (Himedia; India) for 24 h at 35 °C. Afterwards, the turbidity of cell suspension was adjusted to 0.5 on McFarland scale in RPMI medium (1 to 5 × 10^6^ cfu/ml). Then, 200 µL of each *Candida* suspension was inoculated into the wells of a 96-well flat-bottom polystyrene plate. At 24 h, 100 µl of the medium was removed and 100 µl of fresh RPMI 1640 plus 2% glucose was added. After 48 h following incubation, the biofilms were washed twice with sterile PBS to remove non-adherent cells. To fix the biofilm cells, 200 ml methanol was added to all wells. Subsequently, the sessile cells were stained with crystal violet 0.1% (W/v) for 15 min. Finally, 200 μl of ethanol-acetone solution was added to each well. Biofilm biomass was measured with microplate reader (Epoch, Spectrophotometer, USA) at 590 nm. The OD cut-off value (ODc) was calculated as three standard deviations above the mean absorbance of the negative control. The biofilm formation was interpreted as follows: non-biofilm producer: OD ≤ ODc, weak biofilm producer: ODc < OD ≤ 2 × ODc, moderate biofilm producer: 2 × ODc < OD ≤ 4 × ODc, and High biofilm producer: OD > 4 × ODc. The biofilm formation assay was repeated three times for each strain. *C. albicans* ATCC 10261 was used as the control strain.

#### Measurement of biofilm metabolic activity

The mature biofilm viability was assessed by 2, 3-bis (2-methoxy-4-nitro-5-sulfophenyl)-5-[(phenylamino) carbonyl]-2-H tetrazolium hydroxide (XTT) assay [20]. Briefly, 48 h after biofilm formation, the cells were washed twice with sterile PBS. A volume of 100 µL of XTT/menadione solution [0.1 mg/mL XTT and 1 mM menadione, (Sigma Co., USA)] was added to each well containing biofilms. Then, the microplates were incubated in the dark for 3 h at 35° C and finally read in a microtiter plate reader at 490 nm. The metabolic activity of biofilms was interpreted as high biofilm formers (HBF) (OD490 > 0.25 (geometrical mean)), low biofilm formers (LBF) (0.25 < OD490 < 0.05) and non-biofilm former (NBF) (OD490 < 0.05).

#### Biofilm structure by scanning electron microscope

The structure of biofilm were analyzed by scanning electron microscope (SEM) according to Brilhante et al., with modifications [21]. After incubation at 35 °C for 24 h, the planktonic cells were removed and biofilm cells were fixed with glutaraldehyde solution (Sigma-Aldrich, StLouis, MO, USA). After incubation, the biofilms were dehydrated with various dilutions of ethanol (20%, 40%, 60%, 80% and 100%). Finally, network of dense hyphal and yeast cells of *Candida* biofilms was examined with scanning electron microscopy.

#### Molecular detection of virulence genes

Polymerase chain reaction was performed to detect the presence of the virulence gene markers, including HWP1, ALS1, ALS3, ALS5, Sap1, Sap5, and PLB1. Multiple PCR was carried out to detect the HWP1 and ALS1genes; singleplex PCR assay was performed to detect the ALS3, ALS5, Sap1, Sap5, and PLB1 genes. The sequence of virulence factor primers for PCR amplification was shown in Table 1. Finally, the PCR products were confirmed and visualized by gel electrophoresis.

**Table 1:** Sequences of primers of virulence factors for PCR amplification

Primer name	Sequence (5' → 3')	PCR Product size (bP)
HWP1	F: ATG ACT CCA GCT GGT TC	503
	R: TAG ATC AAG AAT GCA GC	
ALS1	F: GAC TAG TGA ACC AAC AAA TAC CAG A	318
	R: CCA GAA GAA ACA GCA GGT GA	
ALS3	F: CCAAGTGTTCCAACAACTGAA	185
	R: GAACCGGTTGTTGCTATGGT	
ALS5	F: TGA CTA CTT CCA GAT TTA TGC CGA G	318
	R: ATT GAT ACT GGT TAT TAT CTG AGG GAG AAA	
PLB	F: CCT ATT GCC AAA CAA GCA TTG TC	179
	R: CCA AGC TAC TGA TTT CAC CTG CTC C	
Sap1	F: GCT CTT GCT ATT GCT TTA TTA	253
	R: CAT CAG GAA CCC ATA AAT CAG	
Sap5	F: AGA ATT TCC CGT CGA TGA GAC TGGT	277
	R: CAA ATT TTG GGA AGT GCG GGA AG	

#### Statistical analysis

The categorical data were described as frequency and percentage and continuous data as mean ± standard deviation (SD). The Kruskal–Wallis test was used for differences in the P_z_, P_rz_ values and biofilm biomass (Bm) between the *Candida* and patient groups. The correlation between these parameters were tested using Spearman’s rank correlation. The geometric mean (GM) MICs, MIC50, MIC90, and MIC ranges were calculated. Statistical analysis was conducted with SPSS version 26 software. *P*-value < 0.05 was considered statistically significant.

## Results

### Oral colonization and distribution of *Candida* species

**Fig. 1:**
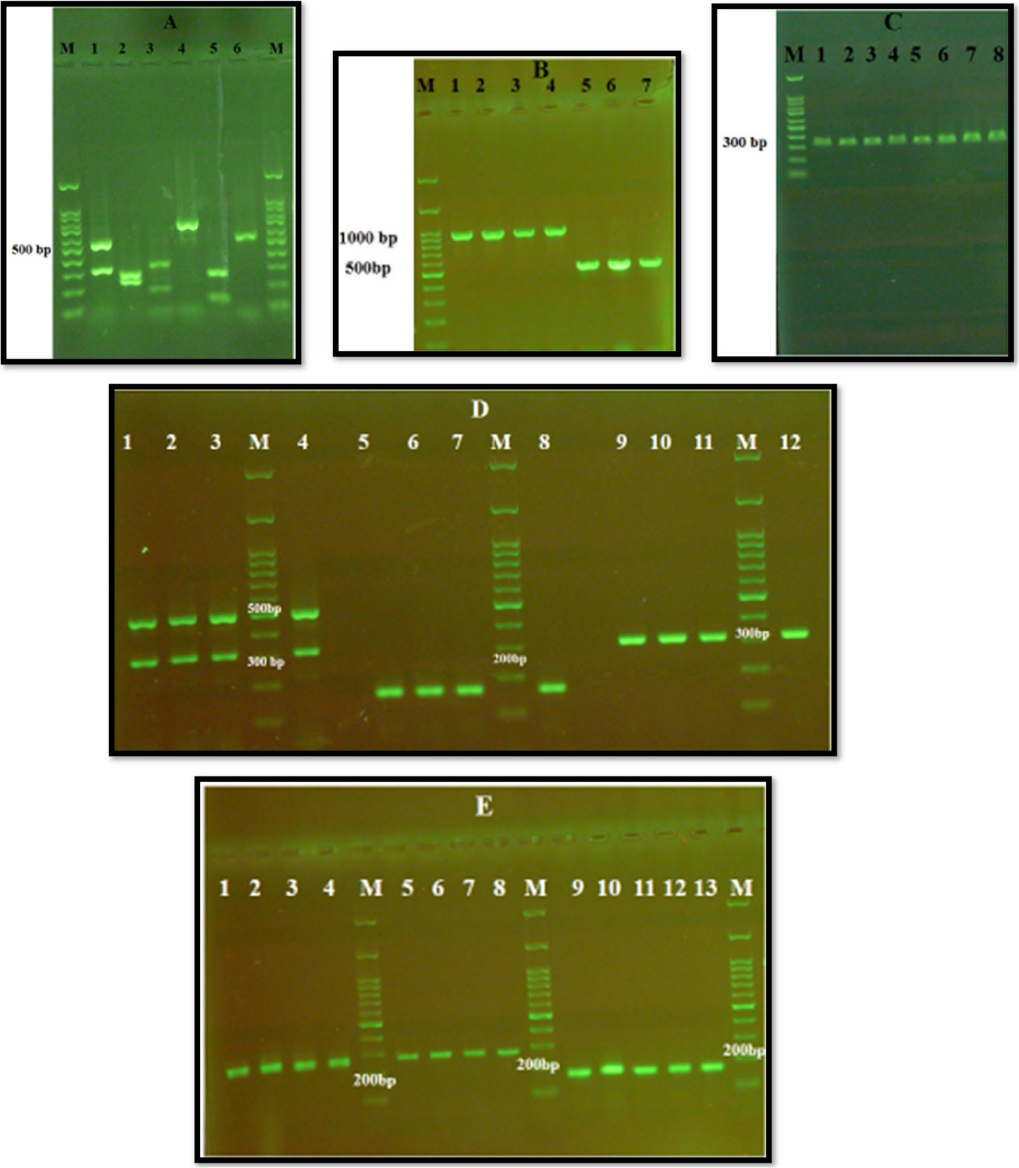
Lane M; 100 bp DNA size marker. **A** Agarose gel electrophoresis of *MspI*-PCR of oral caviry *Candida* species, Lane 1–6; *C. glabrata* (320, 560 bp), *C. albicans* (239, 298 bp)*, C. tropicalis* (186,340 bp)*, C. kefyr* (720 bp), C.lusitaniae (118,264 bp) and *C. parapsilosis* (530 bp)*.* **B** Amplification of the Hwp1 gene, Lane 1–4; *C.albicans* (∼1000 bp), Lane 5–7; *C.dubliniensis* (569 bp). **C** Multiplex PCR of *C. glabrata* complex, Lane 1–8; *C.glabrata* (397 bp). **D** Multiplex PCR of ALS1 (319 bp) and HWP1 (503 bp) genes, Lane 1–4. Singleplex PCR of ALS3 (158bp), Lane 5-8 and ALS5 (318 bp), Lane 9–12. **E** PCR amplified of Sap1 (253 bp); Lane1-4, Sap5 (277 bp); Lane 5–8, PLB1 (179 bp); Lane 9–13

We studied 127 participants with chronic renal failure, including 74 males (58.3%) and 53 females (41.7%), with a mean age of 57 ± 10.5 years, range (27–79 years) (Table 2). The participants were classified into two groups: 69 patients (54.3%) with DM and 58 (45.7%) non-DM patients. The use of dental prostheses was observed in 42% of patients with DM and 26% of non-DM patients. Smoking history was observed in 16% and 24% of DM and non-DM patients, respectively. Oral *Candida* colonization was observed in 47.8% in the hemodialysis patients with DM and 41.4% in non-DM patients. Clinical and microbiological signs and symptoms of oral candidiasis were seen in 18.8% and 7% of DM and non-DM patients, respectively. A statistically significant difference was observed between *Candida* colonization and use of dental prosthesis (*P* < 0.05). A total of 91 *Candida* isolates from oral cavity of hemodialysis patients were identified based on germ tube test, chlamydospore production on cornmeal agar with tween 80, colony color change result from cleavage of chromogenic substrates by species-specific enzymes, and finally by PCR- RFLP technique.. The frequency of *C. albicans* and NAC species in DM and non-DM patients undergoing hemodialysis was 47.5% and 52.5%, respectively. The distribution of the *Candida* species isolated showed that *C. albicans* complex was the most prevalent species (48; 52.7%), followed by *C. glabrata* (15; 16.5%), *C. tropicalis* (11; 12%), *C. kefyr* (8; 8.8%), *C. parapsilosis* (6; 6.7%), and *C. lusitaniae* (3; 3.3%) (Fig. 1a). Among the *C. albicans* complex, three isolates (6.2%) of *C. dubliniensis,* based on the HWP1 gene amplification, were identified, whereas *C. africana* was not noted in the *C. albicans* complex (Fig. 1b). The multiplex PCR of the 5.8-S ribosomal RNA gene showed that all isolates of *C. glabrata* complex with DNA fragment of 397 bp belonged to *C. glabrata *sensu stricto (Fig. 1c). Colonization was observed by one or two *Candida* species in 77% and 23% of hemodialysis patients, respectively.

**Table 2:** Demographic characteristics of 127 patients (diabetic and non-diabetic) with chronic renal failure undergoing hemodialysis

Characteristic	Diabetics (*n* = 66)	Non-diabetics (*n* = 58)	*p* value
Age (years)^a^	58.3 ± 11.5 n (%)	55.3 ± 9.1 N (%)	0.3
**Gender**			
Males	42(61)	32(55.2)	0.51
Females	27(39)	26(44.8)	
**Renal disease**			
Diabetic nephropathy	65(100)	-	
Hypertension	41(59.4)	47(81)	0.09
Glomerulonephritis	-	4(7)	0.03
Others	-	19(32.8)	
DM2 time of diagnosis(years)^a^	19.81 ± 8.16	-	
**Type of dialysis**			
Hemodialysis (HD)	69(100)	58(100)	
Time of dialysis (months)^a^	14.86 ± 3.19	14.79 ± 2.36	0.2
Smoking	11(16)	14(24)	0.24
Use of removable prosthesis	29(42)	15(26)	0.06

^a^Mean ± standard deviation

### Antifungal susceptibility testing

The in vitro antifungal susceptibility profile of five antifungal agents used for planktonic forms of *Candida* species is summarized in Table 3. The antifungal susceptibility activity against planktonic forms showed that all *C. albicans* isolates (100%) were susceptible to AMB, ITC, VRC and CAS with MICs range from 0.032 to 0.125 µg/mL, whereas the resistance rate of *C. albicans* planktonic cells to fluconazole was 6.3% (MIC ≥ 64 μg/mL) with susceptible dose dependent (SDD) of 10.5% (MIC 4 µg/mL) and sensitivity rate of 83.2%. All planktonic forms of non-*albicans Candida* species were susceptible to AMB, FLC, ITC, VRC and CAS, and only one isolate (6.7%) of *C. glabrata* (MIC ≥ 64 μg/mL) was fluconazole-resistant.

**Table 3:** Antifungal susceptibility profile of *Candida* species isolated from oral cavities of patients undergoing hemodialysis

*Candida* species/number of strains	Antifungal	MIC Range	MIC_50_ (µg/mL)	MIC_90_ (µg/mL)	GM
*C.albicans* (*n* = 45)	AMB	0.032–0.125	0.032	0.125	0.053
	CAS	0.032–0.125	0.062	0.125	0.055
	FLC	0.062–64	0.5	4	0.51
	ITC	0.032–0.5	0.062	0.125	0.064
	VRC	0.032–0.125	0.062	0.125	0.055
*C.glabrata* (*n* = 15)	AMB	0.032–0.125	0.125	0.125	0.094
	CAS	0.032–0.062	0.032	0.062	0.043
	FLC	0.5–64	0.5	2	0.45
	ITC	0.062–0.125	0.062	0.125	0.075
	VRC	0.032–0.125	0.032	0.062	0.045
*C.tropicalis* (*n* = 11)	AMB	0.032–0.5	0.125	0.125	0.057
	CAS	0.032–0.125	0.032	0.125	0.067
	FLC	0.032–0.125	0.062	0.125	0.07
	ITC	0.032–0.125	0.062	0.125	0.088
	VRC	0.032–0.062	0.032	0.062	0.041
*C. kefyr* (*n* = 8)	AMB	0.032–0.125	0.032	0.125	0.055
	CAS	0.032–0.032	0.032	0.032	0.057
	FLC	0.062–0.5	0.25	0.5	0.57
	ITC	0.032–0.062	0.032	0.062	0.041
	VRC	0.032–0.062	0.032	0.062	0.041
*C.parapsilosis* (*n* = 6)	AMB	0.032–0.125	0.062	0.125	0.062
	CAS	0.062–0.25	0.25	0.25	0.124
	FLC	0.125–0.25	0.125	0.25	0.25
	ITC	0.032–0.125	0.062	0.125	0.062
	VRC	0.032–0.062	0.032	0.062	0.039
	AMB	0.062–0.062	0.062	0.062	0.062
*C. lusitani* (*n* = 3)	CAS	0.032–0.032	0.032	0.032	0.032
	FLC	0.062–0.125	0. 125	0.125	0.098
	ITC	0.062–0.062	0.062	0.062	0.062
	VRC	0.032–0.062	0.062	0.062	0.049
	AMB	0.062–0.062	0.062	0.062	0.062
*C. dubliniensis* (*n* = 3)	FLC	0.032–0.062	0.062	0.062	0.049
	ITC	0.062–0.062	0.062	0.062	0.062
	VRC	0.032–0.032	0.032	0.032	0.032
	CAS	0.032–0.032	0.032	0.032	0.032

## Phenotypic characterization of virulence factors

### Hydrolytic enzyme production

**Fig. 2:**
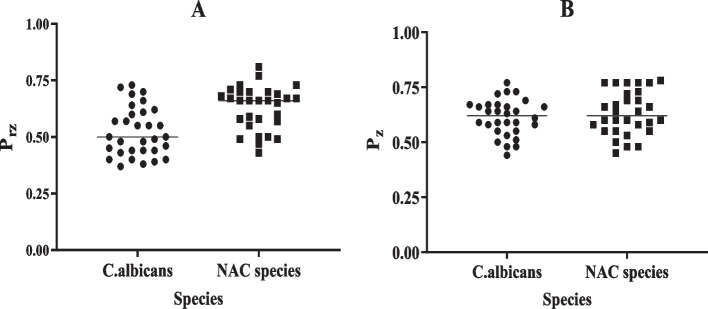
**A**, **B** Comparison of proteinase and phospholipase production of *Candida albicans* and non*-albicans Candida* (NAC) species isolated from oral cavity of hemodialysis patients. There was no statistically significant difference in P_rz_ and P_z_ values between *C. albicans* and NAC groups (*p* > 0.05)

The P_rz_ and P_z_ values of *C.albicans* and NAC species isolated from patients undergoing hemodialysis are shown in Fig. 2. The P_rz_ values for *C. albicans* strains ranged from 0.37 to 0.66 in DM group and 0.44–0.73 in non-diabetic group (*p* < 0.05). The NAC species showed P_rz_ values ranging from 0.43 to 0.77 and 0.55 to 0.81 for DM and non-DM patients, respectively (*p* > 0.05). There was no statistically significant difference in P_rz_ values between *C. albicans* and NAC groups (*p* > 0.05). The mean P_z_ values for *C. albicans* and NAC species from those with DM was 0.61 ± 0.09 and 0.62 ± 0.06, respectively. Also, the average values for phospholipase activity of *C. albicans* and NAC species was 0.62 ± 0.11 and 0.66 ± 0.08 for non-DM group, respectively. There was no statistically significant difference in P_z_ values between *C. albicans* and NAC species groups (*p* > 0.05). In addition, there were no significant differences between the P_rz_ and P_z_ values of isolates and smoking habit or the use of oral denture (*p* > 0.05).

#### Biofilm formation

**Fig. 3:**
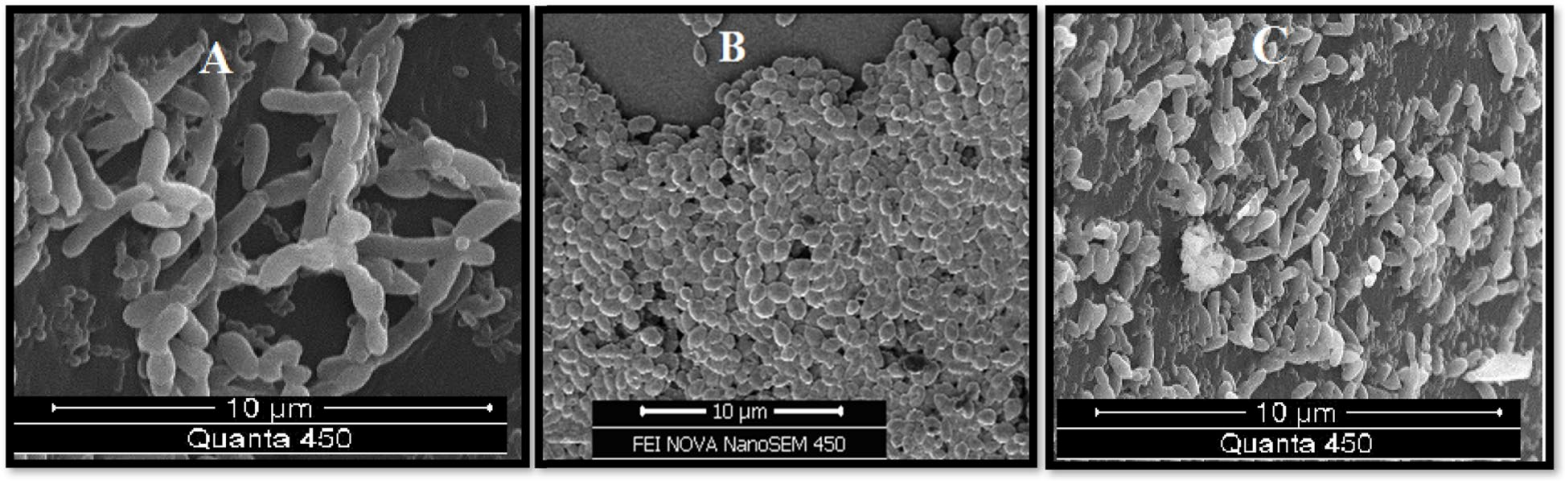
Scanning electron microscopy (SEM) images of the network of biofilm-forming strains of *Candida* species isolated from oral cavities of patients undergoing hemodialysis. **A** biofilm production of *C.albicans* (blastoconidia and pseudohyphae), **B** biofilm production of *C.glabrata* (blastoconidia), **C** biofilm production of *C.tropicalis* (blastoconidia and pseudohyphae). Magnification × 10,000 FEI scanning electron microscope with 10 µm scale

The biofilm biomass (Bm) of clinical *Candida* species is shown in Table 4. The NAC species produced higher level of biomass (average Abs: 0.87 ± 0.51) than *C. albicans* (average Abs: 0.52 ± 0.37) (*P* = 0.001). Among NAC species, *C. tropicalis* showed higher biomass production (average Abs: 1.26 ± 0.53) compared to other NAC group. The biofilm biomass of *Candida* species isolated from the patients with DM was higher than non-DM patients (*p* < 0.05). In addition, there were significant differences between the Bm and use of dental prostheses (*p* < 0.05). The biofilm formation of oral *Candida* species is classified into 3 categories including high biofilm formers (HBF), low biofilm formers (LBF), and non-biofilm formers (NBF) by XTT reduction method which is shown in Table 4. The results showed that biofilm forming ability was present in 66.7% of *C. albicans*, whereas this capability was observed in 88.4% of NAC species. The highest and lowest frequency of HBF was found in *C. tropicalis* and *C. kefyr*, respectively. No significant differences were obtained between the metabolic activity of biofilm and the use of dental prostheses (*p* > 0.05). Furthermore, the results showed a positive correlation between the biofilm biomass and P_rz_ values (*r* = 0.477, *p* = 0.001). The correlation was also statistically significant between the biofilm biomass and the metabolic activity for all strains (*r* = 0.436, *p* = 0.01).. Additionally, a negative correlation was observed between biofilm biomass and FLC MICs FLC (*r* =  − 0.315, *p* < 0.05). The mature *Candida* biofilm with layers of blastoconidia and pseudohyphae was shown by scanning electron microscopy in Fig. 3.

**Table 4:** Biofilms biomass of *Candida* species isolated from oral cavity of hemodialysis patients and the frequency of biofilm forming capacity in different levels

Species	Biofilm (crystal violet)	Biofilm formers (XTT)		
	**Mean ± SD**	**No. of isolates (%)**		
		**HBF**^**1**^	**LBF**^**2**^	**NBF**^**3**^
*C.albicans* (*n* = 45)	0.52 ± 0.37	16 (35.6)	14 (31)	15 (33.4)
*C. dubliniensis* (*n* = 3)	0.41 ± 0.32	1 (33.3)	2 (66.7)	-
NAC species (*n* = 43)	0.87 ± 0.51	21 (48.8)	17 (39.5)	5 (11.6)
*p-value* (*C.albicans* vs NAC)	< 0.001			
*C.glabrate* (*n* = 15)	0.64 ± 0.39	7 (46.7)	6 (40)	2 (13.3)
*C.tropicalis* (*n* = 11)	1.26 ± 0.53	9 (91.8)	2 (18.2)	-
*C. kefyr* (*n* = 8)	0.67 ± 0.51	1 (12.5)	5 (62.5)	2 (25)
*C.parapsilosis* (*n* = 6)	1.07 ± 0.24	4 (66.7)	2 (33.3)	
*C. lusitani* (*n* = 3)	0.69 ± 0.59	-	2 (66.7)	1 (33.3)
Total (*n* = 91)	0.69 ± 0.47	38 (41.8)	33 (36.3)	20 (22)

^1^*HBF* High biofilm formers, ^2^*LBF* Low biofilm formers, ^3^*NBF* Non-biofilm formers

#### Molecular characteristics of virulence genes of *Candida* species

The results of gel electrophoresis for examined genes in *Candida* species are shown in Fig. 1(D,E).The frequency of Als1, ALS3, ALS5, and HWP1 genes was as follows: *C.albicans* (75.6%, 86.7%, 57.8%, and 75.6%), *C. glabrata* (80%, 86.7%, 66.7%, and 80%), *C. tropicalis* (63.6%, 91%, 54.5%, and 81.8%), *C. kefyr* (50%, 75%, 75%, and 62.5%), *C. parapsilosis* (66.7%, 83.3%, 50%, and 66.7%), *C. lusitaniae* (66.7%, 66.7%, 33.3%, and 66.7%), and (33.3%, 100%, 33.3% and 66.7%), respectively. The prevalence of Sap1, Sap5, and PLB1 genes was as follows: *C.albicans* (60%, 82.2%, and 57.8%), *C. glabrata* (66.7%, 73.3%, and 53.3%), *C. tropicalis* (72.7%, 83.3%, and 72.7%), *C. kefyr* (62.5%, 75%, and 37.5%), *C. parapsilosis* (66.7%, 83.3%, and 50%), *C. lusitaniae* (33.3%, and 66.7%, 66.7%) and *C. dubliniensis* (33.3%, 66.7%, and 33.3%), respectively. The most common virulence factors detected were ALS3 and Sap5 genes in the *Candida* isolated from oral infection and colonization. Among biofilm-producing isolates, the highest detected gene was ALS3 (88%), followed by HWP1 (75.8%), ALS1 (71.2%), and ALS5 (57.6%). The prevalence of Sap5 and Sap1 genes among proteinase-producing strains were 82.4% and 64.7%, respectively. The PLB1 gene was detected in 60.7% of phospholipase-positive strains.

## Discussion

The colonization of *Candida* species in the oral mucosa of immunocompromised patients can lead to invasive infections [22–24]. Our results showed that oral *Candida* colonization was observed in 47.8% in the hemodialysis patients with DM and 41.4% in non-DM patients. In previous studies, the frequency of *Candida* colonization in hemodialysis patients has been reported as 39% to 56% [8, 25, 26]. In this study *C. albicans* was the most common species with a frequency of 49.5%. The prevalence of *C. albicans* isolated from the oral mucosa of CKD patients has been reported in Mexico (73.9%) [26], Brazil (63%) [25], and Turkey (51%) [8]. In our study, *C. glabrata* was the most common *Candida* among non-*albicans Candida* species (16.5%) which is similar to other studies performed on chronic dialysis patients: 25% [8], 22% [26], 16% [25], and 12.5% [27]. The antifungal susceptibility profile in our study showed that all *Candida* species (100%) tested was susceptible to AMB, ITC, VRC and CAS agents. Godoy et al., reported that all yeastsisolated from the oral cavities of patients with chronic renal failure undergoing hemodialysis were sensitive to FLC, NYS, AMB, VRC, and CAS agents [25]. Queiroz et al. in Brazil reported that all the species isolated from chronic kidney patients undergoing hemodialysis were sensitive to AMB [28]. In addition, antifungal susceptibility profiles showed that 6.7% and 8.8% of *C. albicans* isolates were FLC-resistant (MIC ≥ 64 μg/mL) and SDD (MIC 4 μg/mL), respectively. Rosa-Garci´a et al. demonstrated that the highest MIC to FLC was 6.7% and 31.8% in *C. albicans* and *C. glabrata* isolated from the oral cavity of hemodialysis patients, respectively [8]. As hemodialysis patients receive several azole antifungal treatments for long periods, the chances of antifungal resistance increase, leading to therapeutic failure in invasive candidiasis infections. Therefore, considering the species distribution and antifungal susceptibility in our study and other similar studies, OC treatment can be a valuable strategy for CKD patients [29–31]. Our findings revealed that the proteinase and phospholipase activities of *Candida* spp. were 71.5% and 59.3%, respectively. Previous studies have reported phospholipase and proteinase activities among 30 to 100% of *Candida* isolates from various sources [32–34]. The role of secretory proteinase as a key hydrolytic enzyme in oral candidiasis is important, and there are reports regarding the relationship between proteinase activity and biofilm formation [35]. In our study, significant differences in the P_rz_ values of the *Candida* spp. were observed between the DM and non-DM groups but with no statistical significance between *C. albicans* and NAC species. In addition, there was an association between protease production and biofilm formation. Considering the role of proteinase, it is possible that the cells within the biofilm network use proteinase to release essential nutrients [35]. In this study, there were no significant differences between the P_z_ values of the *Candida* species in the DM group and the non-DM group. Moreover, there was no correlation between phospholipase activity and biofilm formation. Rajendran et al., showed lack of association between phospholipase and biofilm production by oral *C. albicans* in patients with DM [36]. Nouraei et al., showed that *C. albicans* isolated from the oral cavity of patients with diabetes mellitus had the highest phospholipase activity compared to non-*albicans* isolates [37]. Our study demonstrated that the biomass and metabolic activity of biofilm in NAC species was higher than *C. albicans*. The biofilm biomass measurement using crystal violet method showed that the highest biomass value was within the NAC species and particularly in *C. tropicalis*, followed by *C. parapsilosis*, and *C. glabrata* which was consistent with previous studies [38, 39]. The structure of biofilm in *Candida* species is different, e.g. *C. glabrata* biofilm consists of round blastoconidia in a monolayer arrangement, while the biofilm of *C. parapsilosis* is compact and that of *C. tropicalis* reveals a multilayer arrangement of blastoconidia and pseudohyphae [21, 38, 39]. In addition, biofilm forming ability of *Candida* spp. by XTT reduction activity demonstrated that there were significant differences between the biofilm forming ability in *C. albicans* and NAC species. Many reports show that the biofilm formation of NAC species is higher than that of *C. albicans*, indicating that biofilm formation is an important virulence factor in the colonization of NAC species [19, 40, 41]. The virulence factors, including adhesion to surfaces as the first step in biofilm formation (ALS gene family and HWP1), and production of secreted virulence factors (Sap and PLB) contribute to the pathogenicity of *Candida* species [42]. In our study, among the *Candida* species isolated from oral cavity, *C. albicans* had the highest frequency for ALS3 and Sap5 genes. This result was similar to previous studies in Iraq, Iran and Mexico [43–45]. Dabiri et al., showed that the expression of SAP in *Candida* species plays an important role in *Candida* infections [46]. Our data showed that the prevalence of the Sap5 and Sap1 genes among the proteinase-producing strains were 82.4% and 60.7%, respectively. Also, the PLB1 gene was detected in 60.7% of phospholipase-positive strains. Furthermore, the frequency of various virulence genes in biofilm-forming isolates showed the following values for ALS3 (88%), HWP1 (81.8%), ALS1 (71.2%), and ALS5 (57.6%).

## Conclusion

The awareness on oral lesions, and the reduction of risk factors associated with oral candidiasis such as use of dental prosthesis in CKD patients are essential. Furthermore, it is necessary to identify *C. albicans* and non-*albicans* species, investigate the antifungal susceptibility profile, and understand the pathogenicity mechanisms of *Candida* species. Finally, considering the growing number of antifungal resistance cases, efforts towards designing new therapeutic strategies against virulence factors are of great importance.

## Data Availability

All data analyzed during this study are included in this published article.

## Abbreviations

Bm: Biofilm biomass
P_rz_: Proteinase activity
P_z_: Phospholipase activity
NAC species: Non-*albicans** Candida* species
CKD: Chronic Kidney Disease
OC: Oral candidiasis
DM: Diabetic patients
HWP: Hyphae wall protein
MIC: Minimum Inhibitory Concentrations
FLC: Fluconazole
AMB: Amphotericin B
ITC: Itraconazole
VRC: Voriconazole
CAS: Caspofungin
